# Statistical inference with joint progressive censoring for two populations using power Rayleigh lifetime distribution

**DOI:** 10.1038/s41598-023-30392-7

**Published:** 2023-03-07

**Authors:** Ahlam H. Tolba, Tahani A. Abushal, Dina A. Ramadan

**Affiliations:** 1 Department of Mathematics, Faculty of Science, Mansoura University, Mansoura, 33516 Egypt; 2 Department of Mathematical Science, Faculty of Applied Science, Umm AL-Qura University, Makkah, Saudi Arabia

**Keywords:** Health care, Risk factors, Mathematics and computing

## Abstract

In this study, point and interval estimations for the power Rayleigh distribution are derived using the joint progressive type-II censoring technique. The maximum likelihood and Bayes methods are used to estimate the two distributional parameters. The estimators’ approximate credible intervals and confidence intervals have also been determined. The Markov chain Monte Carlo (MCMC) method is used to provide the findings of Bayes estimators for squared error loss and linear exponential loss functions. The Metropolis–Hasting technique uses Gibbs to generate MCMC samples from the posterior density functions. A real data set is used to show off the suggested approaches. Finally, in order to compare the results of various approaches, a simulation study is performed.

## Introduction

**Figure 1:**
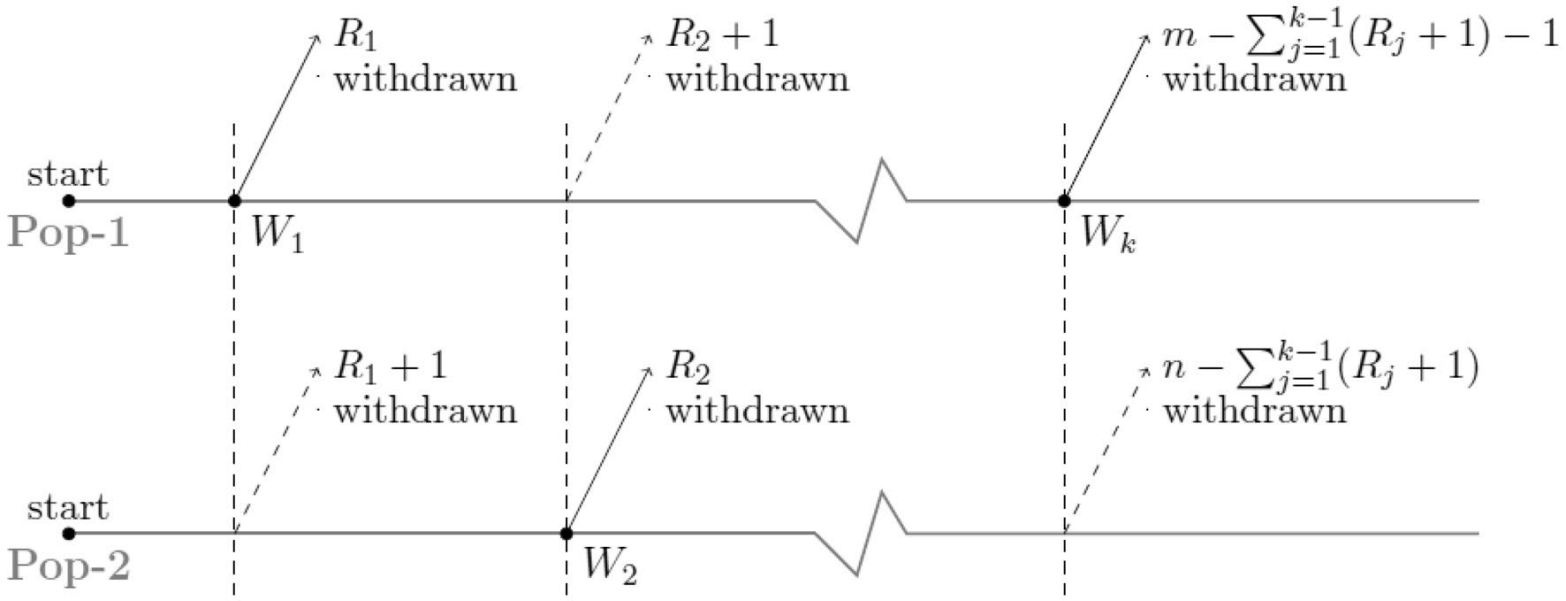
Case-I: kth failure comes from Pop-1.

**Figure 2:**
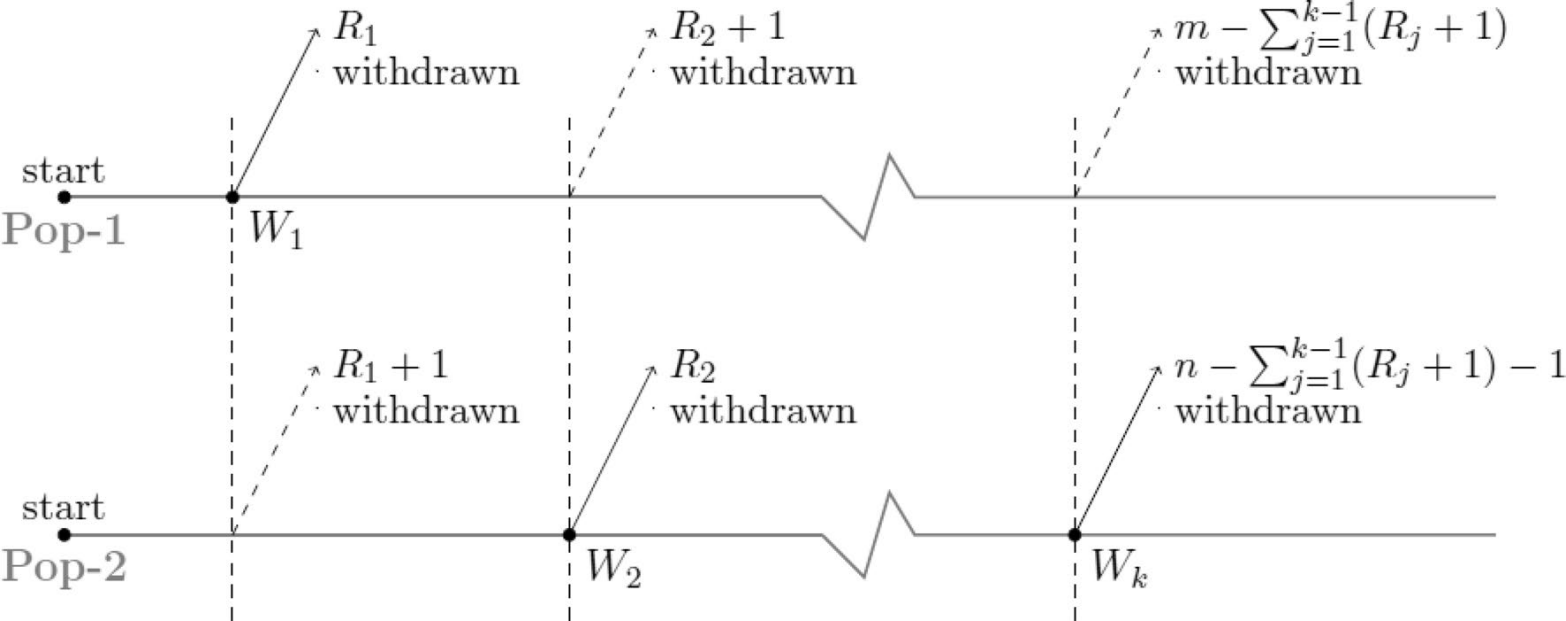
Case-II: kth failure comes from Pop-2.

The joint censoring method is extremely advantageous and practical when conducting comparative life tests of products from different units inside the same facility. Assume that two different lines within the same facility are producing products. Assume that two independent samples of sizes *m* and *n* are chosen at random from these two production lines and placed in a life-testing experiment at the same time. The experimenter uses a combination progressive type-II censoring strategy to save time, money, and the life-testing is completed when a specified number of failures occur to see:^1–4^, and^5^. In the literature, many authors have looked at the joint progressive type II censoring scheme (JP-II-CS) and inference methods. For example^6^ used the joint progressive type II censoring scheme (JP-II-CS) to incorporate the likelihood inference of two exponential distributions^7^ investigated Bayes estimation with JP-II-CS and the LINEX loss function^8^ provided the likelihood inference for *k* exponential distributions under the JP-II-CS^9^ introduced Weibull parameter point and interval estimates based on JP-II-CS. The JP-II-CS of two populations was considered by^10^, because the lifetime distributions of the experimental units in both populations follow two-parameter generalised exponential distributions and^11^ introduced the statistical inference of inverted exponentiated Rayleigh distribution under joint progressively type-II censoring. Also,^12^ proposed the power Rayleigh distribution, which has been utilised for lifetime modelling in reliability analysis,^13^ the lifetime performance index with power Rayleigh distribution is estimated with progressive first-failure censoring,^14^ presented methods for simulating the parameter of the Akshaya distribution using Bayesian and Non-Bayesian estimation,^15^ introduced a new distribution called generalized power Akshaya distribution and its applications,^16^ discussed characteristics and applications of the extended Cosine generalized family of distributions for reliability modeling, and^17^ developed a novel, flexible modification of the log-logistic distribution to model the COVID-19 mortality rate. It has also been fitted using a wide range of observational data from a variety of fields, including meteorology, finance, and hydrology (see^18^). Moreover,^19^ discussed an application of type II half logistic Weibull distribution inference for reliability analysis with bladder cancer. The joint progressive censoring scheme is quite useful to compare the lifetime distribution of products from different units which are being manufactured by two different lines in the same facility. The joint progressive censoring (JPC) scheme introduced by Rasouli and Balakrishnan^6^ can be briefly stated as follows. It is assumed that two samples of products of sizes m and n, respectively, are selected from these two lines of operation (say Line 1 and Line 2) for two populations Pop-1 and Pop-2 as shown in Figs. 1 and 2, and they are placed on a life testing experiment simultaneously.

With application to flood frequency analysis, the power Rayleigh distribution has extremes. JP-II-CS is described as follows by^20^. The family of Rayleigh distribution is formed such as generalized Rayleigh distribution is introduced by^12^ and ^21,22^ discussed the log Rayleigh distribution^23^, derived beta generalized Rayleigh distribution, Weibull Rayleigh distribution is introduced by^24^ and ^25^ introduced exponentiated Rayleigh distribution. Several authors have considered extensions of Rayleigh distribution such as inverse Rayleigh by^26^, weighted inverse Rayleigh distribution by^27^ and transmuted Rayleigh distribution by^28^. The quality of the procedures used in statistical analysis depends heavily on the assumed probability model or distribution. Let $X_{1},X_{2},...., X_{m}$ represent the lifetimes of *m* units for product A, and they are considered to be independent and identically distributed (iid) random variables from the power Rayleigh distribution with a cumulative distribution function (cdf) of

1 $$\begin{aligned} F(x; \alpha _{1}, \beta _{1})= 1- e^{\frac{-x^{2\beta _{1}}}{\alpha _{1}^{2}}}, \ \ \ \ \ \ \ \ \ \ x>0, \alpha _{1}, \beta _{1}>0, \end{aligned}$$

and probability density function (pdf) is

2 $$\begin{aligned} f(x; \alpha _{1}, \beta _{1})=\frac{\beta _{1}}{\alpha _{1}^{2}} x^{2\beta _{1}-1} e^{\frac{-x^{2\beta _{1}}}{\alpha _{1}^{2}}}, \ \ \ \ \ \ \ \ \ x>0, \alpha _{1}, \beta _{1}>0. \end{aligned}$$

Similarly, let $Y_{1},Y_{2},...., Y_{n}$, are lifetimes of *n* units for product B, and they are supposed to iid random variables from the power Rayleigh distribution with cdf is given by

3 $$\begin{aligned} G(x; \alpha _{2}, \beta _{2})= 1- e^{-\frac{x^{2\beta _{2}}}{\alpha _{2}^{2}}}, \ \ \ \ \ \ \ \ \ \ y>0, \alpha _{2}, \beta _{2}>0, \end{aligned}$$

and probability density function (pdf) is

4 $$\begin{aligned} g(x; \alpha _{2}, \beta _{2})=\frac{\beta _{2}}{\alpha _{2}^{2}} x^{2\beta _{2}-1} e^{-\frac{x^{2\beta _{2}}}{\alpha _{2}^{2}}}, \ \ \ \ \ \ \ \ \ \ y>0, \alpha _{2}, \beta _{2}>0, \end{aligned}$$

where $\beta _{1}$ and $\beta _{2}$ are the shape parameters and $\alpha _{1}$ and $\alpha _{2}$ are a scale parameters. Let $K = m+n$ denotes the total sample size and $\lambda _{1}\le \lambda _{2} \le ...\le \lambda _{K}$ indicates the order statistics of the *K* random variables ${X_{1},X_{2},...., X_{m},Y_{1},Y_{2},...., Y_{n} }$. The JP-II-CS is applied as follows. At the time of the first failure, $R_{1}$ units are randomly removed from the remaining $K - 1$ surviving units. Similarly, at the time of the second failure, $R_{2}$ units are randomly withdrawn from the remaining $K - R_{1} - 2$ surviving units, etc. In the end, at the time of the *r*th failure units, all remaining $R_{r} = K- r -\sum _{i=1}^{r-1} R_{i}$ surviving units are withdrawn from the life-testing experiment. Where the JP-II-CS $(R = R_1, R_2,...,R_r)$ and the total number of failures *r* are prefixed before the experiment. Suppose that $R_i = S_i + T_i, i = 1, ..., r$ and $S_i$ and $T_i$ indicate the number of units withdrawn at the time of the *i*th failure is related to *X* and *Y* samples respectively, and these are unknown and random variables. The data observed in this form will consist of $(H, \lambda , S)$, where $(H = H_1, H_2,...,H_r)$, $H_i = 1 \ or \ 0$ if $\lambda _i$ comes from *X* or *Y* failure, respectively, $(\lambda = \lambda _1, \lambda _2,...,\lambda _r)$ with $r < K$, and $(S = S_1, S_2,...,S_r)$.

In this research, the lifetime distributions of the experimental units in the two populations follow two-parameter generalized exponential distributions with the same scale parameter but different shape parameters. We investigate both the likelihood and the Bayesian inference of unknown model parameters. By solving a three-dimensional optimization problem, the maximum likelihood estimators (MLEs) of the unknown parameters can be produced. This problem can be solved using the Newton-Raphson approach. In this instance, the Hessian matrix must be computed, which may not be in the most convenient format. Furthermore, it has been discovered that the traditional Newton-Raphson approach may not be suitable for small effective sample sizes.

The following is a list of the paper’s objectives: The maximum likelihood estimators (MLEs) of the power Rayleigh distribution’s unknown parameters are derived in Maximum likelihood estimation” section. Approximate confidence intervals (ACIs) based on the MLEs are presented in “Bayesian method” section. “Application of real data” section is where the Bayesian analysis is carried out. In “Simulation” section, we examine real data sets to demonstrate the estimating methods presented in this paper. In “Conclusion” section, the simulation results are shown. Section 8 concludes with a brief conclusion.

## Maximum likelihood estimation

Assume that $X_1, X_2,... X_m$ are independently and identically distributed (i.i.d.) power Rayleigh random variables representing the lifetimes of *m* units for product A. Similarly, $Y_1, Y_2,...$, and $Y_n$ are assumed to denote the lifetimes of *n* units for product B, and they are assumed to be independent and identically distributed (i.i.d.) power Rayleigh random variables. According to Rasouli and Balakrishnan^6^, the likelihood function of$(S,H,\lambda )$ can be written as follows

5 $$\begin{aligned} L(\alpha _{1},\alpha _{2}, \beta _{1}, \beta _{2}; H, \lambda ,S)= & {} C\prod \limits _{i=1}^{r}\left[ \left( f(\lambda _{i})\right) ^{h_{i}} \left( g(\lambda _{i})\right) ^{1-h_{i}}\right] \left[ \left( \bar{F}(\lambda _{i})\right) ^{S_{i}} \left( \bar{G}(\lambda _{i})\right) ^{t_{i}}\right] , \end{aligned}$$

where $\lambda _{1}\le \lambda _{2}\le ...\le \lambda _{r},$ $\bar{F}=1-F,$ $\bar{G}=1-G,$ $\sum _{i=1}^{r} s_{i} = m-m_{r},$ $\sum _{i=1}^{r} t_{i} = n-n_{r},$ $\sum _{i=1}^{r} R_{i} = \sum _{i=1}^{r} s_{i} + \sum _{i=1}^{r} t_{i}$, and $C=D_{1} D_{2}$ with

6 $$\begin{aligned} D_{1}= & {} \prod \limits _{j=1}^{r}{\left( m-\sum _{i=1}^{j-1} h_{i}-\sum _{i=1}^{j-1} s_{i}\right) h_{j}+\left( n-\sum _{i=1}^{j-1} (1-h_{i})-\sum _{i=1}^{j-1} t_{i}\right) (1-h_{j})},\nonumber \\ D_{2}= & {} \prod \limits _{j=1}^{r}\left( \frac{\left( {\begin{array}{c}m-\sum _{i=1}^{j-1}h_{i}-\sum _{i=1}^{j-1}s_{i}\\ s_{i}\end{array}}\right) \left( {\begin{array}{c}n-\sum _{i=1}^{j-1}(1- h_{i})-\sum _{i=1}^{j-1}t_{i}\\ t_{i}\end{array}}\right) }{\left( {\begin{array}{c} m+n-j-\sum _{i=1}^{j-1} R_{i}\\ R_{j}\end{array}}\right) }\right) .\nonumber \\ L(\alpha _{1},\alpha _{2}, \beta _{1}, \beta _{2})= & {} C \left( \frac{\beta _{1}}{\alpha _{1}^2}\right) ^{m_{r}} \left( \frac{\beta _{2}}{\alpha _{2}^2}\right) ^{n_{r}} \prod \limits _{i=1}^{r}\lambda _{i}^{(2\beta _{1}-1)h_{i}}e^{\frac{-h_{i} \lambda _{i}^{2\beta _{1}}}{2\alpha _{1}^{2}}} \lambda _{i}^{(2\beta _{2}-1)(1-h_{i})}e^{\frac{-(1-h_{i})\lambda _{i}^{2\beta _{2}-1}}{2\alpha _{1}^{2}}} e^{\frac{-s_{i}\lambda _{i}^{2\beta _{1}}}{\alpha _{1}^{2}}}e^{\frac{-t_{i}\lambda _{i}^{2\beta _{2}}}{\alpha _{2}^{2}}}. \end{aligned}$$

As a result, the log-likelihood function can be written as:

7 $$\begin{aligned} \ell (\alpha _{1},\alpha _{2}, \beta _{1}, \beta _{2}; H, \lambda ,S)= & {} m_{r}\log \beta _{1}-2 m_{r}\log \alpha _{1}+ n_{r}\log \beta _{2}-2 n_{r}\log \alpha _{2} +\sum _{j=1}^{r}(2\beta _{1}-1)h_{i}\log \lambda _{i}\nonumber \\- & {} \sum _{i=1}^{r}\frac{h_{i}\lambda _{i}^{2\beta _{1}}}{2\alpha _{1}^{2}} +\sum \limits _{i=1}^{r}(2\beta _{2}-1)(1-h_{i})\log \lambda _{i}-\sum \limits _{i=1}^{r} \frac{(1-h_{i})\lambda _{i}^{2\beta _{2}-1}}{2\alpha _{2}^{2}}\nonumber \\- & {} \sum _{i=1}^{r}\frac{s_{i}\lambda _{i}^{2\beta _{1}}}{\alpha _{1}^{2}}-\sum _{i=1}^{r} \frac{t_{i}\lambda _{i}^{2\beta _{2}}}{\alpha _{2}^{2}}. \end{aligned}$$

To estimate the unknown parameters, take the first derivative of Eq. (7) with respect to $\alpha _{1},\alpha _{2}, \beta _{1}$, and $\beta _{2}$, which are given by:

8 $$\begin{aligned} \frac{\partial \ell }{\partial \alpha _{1} }= & {} \frac{-2m_{r}}{\alpha _{1} }+\sum \limits _{i=1}^{r}\frac{h_{i}\lambda _{i}^{2\beta _{1}}}{ \alpha _{1}^{3}}+2\sum \limits _{i=1}^{r}\frac{s_{i}\lambda _{i}^{2\beta _{1}}}{ \alpha _{1}^{3}}, \end{aligned}$$

9 $$\begin{aligned} \frac{\partial \ell }{\partial \alpha _{2} }= & {} \frac{-2n_{r}}{\alpha _{2} }+\sum \limits _{i=1}^{r}\frac{(1-h_{i})\lambda _{i}^{2\beta _{2}-1}}{ \alpha _{2}^{3}}+2\sum \limits _{i=1}^{r}\frac{t_{i}\lambda _{i}^{2\beta _{2}}}{ \alpha _{2}^{3}}, \end{aligned}$$

10 $$\begin{aligned} \frac{\partial \ell }{\partial \beta _{1} }= & {} \frac{m_{r}}{\beta _{1} }+2\sum \limits _{i=1}^{r}h_{i}\log \lambda _{i} -2 \sum \limits _{i=1}^{r}\frac{s_{i}\lambda _{i}^{2\beta _{1}}}{ \alpha _{1}^{2}}\log \lambda _{i}, \end{aligned}$$

and

11 $$\begin{aligned} \frac{\partial \ell }{\partial \beta _{2} }=\frac{n_{r}}{\beta _{2} }+2\sum \limits _{i=1}^{r}(1-h_{i})\log \lambda _{i} -2 \sum \limits _{i=1}^{r}\frac{t_{i}\lambda _{i}^{2\beta _{2}-1}}{ \alpha _{2}^{2}}\log \lambda _{i}. \end{aligned}$$

The system of normal equations $\frac{\partial \ell }{\partial \alpha _{1} }=0$, $\frac{\partial \ell }{\partial \alpha _{2} }=0$, $\frac{\partial \ell }{\partial \beta _{1} }=0$, and $\frac{\partial \ell }{\partial \beta _{2} }=0$ has not closed form of its solution, so the numerical techniques to estimate the unknown parameters $\alpha _{1},\alpha _{2}, \beta _{1}$, and $\beta _{2}$ are used.

### Asymptotic confidence intervals

The maximum likelihood estimators for the parameters cannot be obtained in analytic form. Therefore, their actual distributions cannot be derived. However, we can use the asymptotic distribution of the maximum likelihood estimator to derive confidence intervals for the unknown parameters $\alpha _{1},\alpha _{2}, \beta _{1}$, and $\beta _{2}$.

The $100(1-\gamma )\%$ CIs for $\alpha _{1},\alpha _{2}$ $\beta _{1}~$, and $\beta _{2}~$ can be calculated using the asymptotic normality of the maximum likelihood estimators with Var($\hat{\alpha _{1}}_{ML}$), Var($\hat{\alpha _{2}}_{ML}$), Var($\hat{\beta _{1}}_{ML}$), and Var($\hat{\beta _{2}}_{ML}$). The second derivatives with respect to $\alpha _{1},\alpha _{2}$, $\beta _{1}~$, and $\beta _{2}~$ are provided by the log-likelihood function in Eq. (7).

$$\begin{aligned} \frac{\partial ^{2}\ell }{\partial \alpha _{1}^{2}}&=\frac{2m_{r}}{\alpha _{1}^{2} }-3\sum \limits _{i=1}^{r}\frac{h_{i}\lambda _{i}^{2\beta _{1}}}{ \alpha _{1}^{4}}-6\sum \limits _{i=1}^{r}\frac{s_{i}\lambda _{i}^{2\beta _{1}}}{ \alpha _{1}^{4}}, \\ \frac{\partial ^{2}\ell }{\partial \alpha _{1}\partial \beta _{1}}&=2\sum \limits _{i=1}^{r}\frac{h_{i}\lambda _{i}^{2\beta _{1}} \log \lambda _{i}}{ \alpha _{1}^{3}}+4\sum \limits _{i=1}^{r}\frac{s_{i}\lambda _{i}^{2\beta _{1}}\log \lambda _{i}}{ \alpha _{1}^{3}}, \\ \frac{\partial ^{2}\ell }{\partial \alpha _{2}^{2}}&=\frac{2n_{r}}{\alpha _{2}^{2} }-3\sum \limits _{i=1}^{r}\frac{(1-h_{i})\lambda _{i}^{2\beta _{2}-1}}{ \alpha _{2}^{4}}-6\sum \limits _{i=1}^{r}\frac{t_{i}\lambda _{i}^{2\beta _{2}}}{ \alpha _{2}^{4}}, \\ \frac{\partial ^{2}\ell }{\partial \alpha _{2}\partial \beta _{2}}&=2\sum \limits _{i=1}^{r}\frac{(1-h_{i})\lambda _{i}^{2\beta _{2}-1} \log \lambda _{i}}{ \alpha _{1}^{3}}+2\sum \limits _{i=1}^{r}\frac{t_{i}\lambda _{i}^{2\beta _{2}}\log \lambda _{i}}{ \alpha _{2}^{3}},\\ \frac{\partial ^{2}\ell }{\partial \beta _{1}^{2}}&=\frac{-m_{r}}{\beta _{1}^{2} }-4\sum \limits _{i=1}^{r}\frac{s_{i}\lambda _{i}^{2\beta _{1}}(\log \lambda _{i})^{2}}{ \alpha _{1}^{2}}, \\ \frac{\partial ^{2}\ell }{\partial \beta _{1}\partial \alpha _{1}}&=4\sum \limits _{i=1}^{r}\frac{s_{i}\lambda _{i}^{2\beta _{1}}\log \lambda _{i}}{ \alpha _{1}^{3}}, \\ \frac{\partial ^{2}\ell }{\partial \beta _{2}^{2}}&=\frac{-n_{r}}{\beta _{2}^{2} }-4\sum -4\sum \limits _{i=1}^{r}\frac{t_{i}\lambda _{i}^{2\beta _{2}}(\log \lambda _{i})^{2}}{ \alpha _{2}^{2}}, \\ \frac{\partial ^{2}\ell }{\partial \beta _{2}\partial \alpha _{2}}&=4\sum \limits _{i=1}^{r}\frac{t_{i}\lambda _{i}^{2\beta _{2}}\log \lambda _{i}}{ \alpha _{2}^{3}},\\ \frac{\partial ^{2}\ell }{\partial \alpha _{1}\alpha _{2}}&=0, \\ \frac{\partial ^{2}\ell }{\partial \partial \alpha _{2}\alpha _{1}}&=0, \\ \frac{\partial ^{2}\ell }{\partial \partial \beta _{1}\beta _{2}}&=0, \\ \frac{\partial ^{2}\ell }{\partial \beta _{1}\partial \beta _{2}}&=0. \end{aligned}$$

Therefore, the observed Fisher information matrix $\hat{I}_{ij}=$ $E\left[ -\partial ^{2}\ell /\partial \phi _{i}~\partial \phi _{j}\right]$, where $i,j=1,2,3,4~$, and  $\phi =\left( \phi _{1},\phi _{2},\phi _{3}, \phi _{4}\right) =\left( \alpha _1,\alpha _2,\beta _{1}, \beta _{2}\right) .$

Hence, the observed information matrix is given by

$$\begin{aligned} \hat{I}\left( \alpha _{1},\alpha _{2},\beta _{1}, \beta _{2}\right) = \begin{pmatrix} -\frac{\partial ^{2}\ell }{\partial \alpha _{1}^{2}} &{} -\frac{\partial ^{2}\ell }{ \partial \alpha _{1}\partial \alpha _{2}} &{} -\frac{\partial ^{2}\ell }{\partial \alpha _{1}\partial \beta _{1}} &{} -\frac{\partial ^{2}\ell }{\partial \alpha _{1}\partial \beta _{2}} \\ -\frac{\partial ^{2}\ell }{\partial \alpha _{2}\partial \alpha _{1}} &{} -\frac{\partial ^{2}\ell }{\partial \alpha _{2}^{2}} &{} -\frac{\partial ^{2}\ell }{\partial \alpha _{2}\partial \beta _{1}} &{} -\frac{\partial ^{2}\ell }{\partial \alpha _{2}\partial \beta _{2}} \\ -\frac{\partial ^{2}\ell }{\partial \beta _{1}\partial \alpha _{1}} &{} -\frac{\partial ^{2}\ell }{\partial \beta _{1}\partial \alpha _{2}} &{} -\frac{\partial ^{2}\ell }{ \partial \beta _{1}^{2}}&{}-\frac{\partial ^{2}\ell }{\partial \beta _{1}\partial \beta _{2}}\\ -\frac{\partial ^{2}\ell }{\partial \beta _{2}\partial \alpha _{1}} &{} -\frac{\partial ^{2}\ell }{\partial \beta _{2}\partial \alpha _{2}} &{} -\frac{\partial ^{2}\ell }{ \partial \beta _{2}\beta _{1}}&{}-\frac{\partial ^{2}\ell }{\partial \beta _{2}^{2}} \end{pmatrix}. \end{aligned}$$

Therefore, the inverting the observed information matrix $\hat{I}\left( \alpha _1,\alpha _2,\beta _{1}, \beta _{2}\right)$ is used to obtain the asymptotic variance-covariance matrix for the MLEs. Where $\hat{I}^{-1}\left( \alpha _1,\alpha _2,\beta _{1}, \beta _{2}\right)$ is obtained by

$$\begin{aligned} \hat{I}^{-1}\left( \alpha _1,\alpha _2,\beta _{1},\beta _{2}\right) =\left( \begin{array}{cccc} \widehat{var(\alpha _{1})} &{} \quad cov(\alpha _{1},\alpha _{2}) &{} \quad cov(\alpha _{1},\beta _{1}) &{} \quad cov(\alpha _{1},\beta _{2}) \\ cov(\alpha _{2},\alpha _{1}) &{} \quad \widehat{var(\alpha _{2})} &{} \quad cov(\alpha _{2},\beta _{1}) &{} \quad cov(\alpha _{2},\beta _{2}) \\ cov(\beta _{1},\alpha _{1}) &{} \quad cov(\beta _{1},\alpha _{2}) &{} \quad \widehat{var(\beta _{1})} &{} \quad cov(\beta _{1},\beta _{2})\\ cov(\beta _{2},\alpha _{1}) &{} \quad cov(\beta _{2},\alpha _{2}) &{} \quad cov(\beta _{2},\beta _{1}) &{} \quad \widehat{var(\beta _{2})} \end{array} \right) . \end{aligned}$$

Thus, the $100(1-\gamma )\%$ normal approximate CIs for $\left( \alpha _1,\alpha _2,\beta _{1}, \beta _{2}\right)$ are

12 $$\begin{aligned} \widehat{\alpha _{1}}\pm Z_{\frac{\gamma }{2}}\sqrt{\widehat{var(\alpha _{1})}},\ \widehat{\alpha _{2}}\pm Z_{\frac{\gamma }{2}}\sqrt{\widehat{var(\alpha _{2})}},\ \widehat{\beta _{1}} \ \pm Z_{\frac{\gamma }{2}}\sqrt{\widehat{var(\beta _{1} )}} \ \text { and }\widehat{\beta _{2}} \ \pm Z_{\frac{\gamma }{2}}\sqrt{\widehat{var(\beta _{2} )}}. \end{aligned}$$

where $Z_{\frac{\gamma }{2}}$ is the percentile of the standard normal distribution with right-tail probability $\frac{\gamma }{2}$.

We will introduce another method to estimate the unknown parameters, such as the Bayesian technique. Bayesian analysis is a successful tool that has been proposed to estimate the unknown parameters. Comparing Bayesian inference to other methods of reasoning has various benefits.

## Bayesian method

This section contains the Bayesian estimates of the unknown parameters $\alpha _{1},\ \alpha _{2}, \beta _{1}$, and $\beta _{2}$ of the power Rayleigh distribution based on JP-II-CS. Prior knowledge has been incorporated in terms of some prior distributions, and here we assume that the four parameters $\alpha _{1},\ \alpha _{2}$, $\ \beta _{1}$, and $\beta _{2}$  are random variables having independent gamma priors.

$$\begin{aligned} \begin{array}{ll} \pi _{1}\left( \alpha _{1}\right) \propto \alpha _{1}^{a_{1}-1}e^{-b_{1}\alpha _{1}}, &{}\quad \quad \alpha _{1}>0, a_{1}, b_{1}>0, \\ \pi _{2}\left( \alpha _{2}\right) \propto \alpha _{2}^{a_{2}-1} e^{-b_{2}\alpha _{2}},&{}\quad \quad \alpha _{2}>0, a_{2}, b_{2}>0, \\ \pi _{3}\left( \beta _{1} \right) \propto \beta _{1}^{a_{3}-1}e^{-b_{3}\beta _{1} },&{}\quad \quad \beta _{1}>0, a_{3}, b_{3}>0, \\ \pi _{4}\left( \beta _{2} \right) \propto \beta _{2}^{a_{4}-1}e^{-b_{4}\beta _{2} },&{}\quad \quad \beta _{2}>0, a_{4}, b_{4}>0, \end{array} \end{aligned}$$

where $a_i, b_i, i=1,2,3,4$ are considered to be known and chosen to indicate the previous assumption on the unknown parameters. As a result, the joint prior density is given as

13 $$\begin{aligned} \pi \left( \alpha _{1},\ \alpha _{2},\ \beta _{1}, \ \beta _{2} \right) = \alpha _{1}^{a_{1}-1}\alpha _{2}^{a_{2}-1}\beta _{1}^{a_{3}-1}\beta _{2}^{a_{4}-1}e^{-b_{1} \alpha _{1}-b_{2}\alpha _{2}-b_{3}\beta _{3}-b_{4}\beta _{4}}. \end{aligned}.$$

The posterior distribution of parameters $\alpha _{1},\ \alpha _{2}~, \beta _{1}$, and $\ \beta _{2}$ indicates $\pi ^{*}\left( \alpha _{1},\ \alpha _{2},\ \beta _{1}, \ \beta _{2} \mid H, \lambda , S\right) ~$ by combining the likelihood function Eq. (6) with the prior via Bayes’ theorem, proportionality can be achieved and it can be written as

14 $$\begin{aligned}{} & {} \pi ^{*}\left( \alpha _{1},\ \alpha _{2},\ \beta _{1}, \ \beta _{2} \mid H, \lambda , S\right) = \nonumber \\{} & {} \quad =\frac{ \pi \left( \alpha _{1},\ \alpha _{2},\ \beta _{1},\beta _{2} \right) ~L(\alpha _{1},\ \alpha _{2},\ \beta _{1}, \beta _{2} \mid H, \lambda , S)}{ \int \limits _{0}^{\infty }\int \limits _{0}^{\infty }\int \limits _{0}^{\infty }\int \limits _{0}^{\infty }\pi _{1}\left( \alpha _{1}\right) ~\pi _{2}\left( \alpha _{2}\right) ~\pi _{3}\left( \beta _{1} \right) ~\pi _{4}\left( \beta _{2} \right) ~L(\alpha _{1},\ \alpha_{2},\ \beta_{1}, \beta _{2} \mid H, \lambda, S)~d\alpha_{1}d\alpha_{2}d\beta_{1}d\beta _{2}}. \end{aligned}$$

From Eq. (14) can be used to evaluate the joint posterior to proportionality.

15 $$\begin{aligned}{} & {} \pi ^{*}\left( \alpha _{1},\alpha _{2},\beta _{1}, \beta _{2} \mid H, \lambda , S\right) \propto \alpha _{1}^{-2m_{r}+a_{1}-1}\alpha _{2}^{-2n_{r}+a_{2}-1}\beta _{1}^{m_{r} +a_{3}-1}\beta _{2}^{n_{r}+a_{4}-1}e^{-b_{1} \alpha _{1}- b_{2}\alpha _{2}-b_{3}\beta _{1}-b_{4}\beta _{2}}\nonumber \\{} & {} \quad \prod \limits _{i=1}^{r}\lambda _{i}^{(2\beta _{1}-1)h_{i}}e^{-\sum _{i=1}^{r}\frac{h_{i}\lambda _{i}^{2\beta _{1}}}{2\alpha _{1}^{2}}} \prod \limits _{i=1}^{r}\lambda _{i}^{(2\beta _{2}-1)(1-h_{i})}e^{-\sum _{i=1}^{r}\frac{(1-h_{i})\lambda _{i}^{2\beta _{2}}}{2\alpha _{2}^{2}}}\nonumber \\{} & {} \quad e^{-\sum _{i=1}^{r}\frac{s_{i}\lambda _{i}^{2\beta _{1}}}{\alpha _{1}^{2}}} e^{-\sum _{i=1}^{r}\frac{t_{i} \lambda _{i}^{2\beta _{2}}}{\alpha _{2}^{2}}}. \end{aligned}$$

We highlighted that solving Eq. (15) analytically is impossible due to the difficulty in obtaining closed forms for the marginal posterior distributions for each parameter. As a result, we propose using the Markov chain Monte Carlo (MCMC) technique to approximate^29^ and generate samples from posterior distributions, as well as to evaluate Bayes estimators of unknown parameters and construct the corresponding CRIs, using squared error (SE) and linear exponential (LINEX) loss functions. Abushal et al.^30^, EL-Sagheer and Hasaballah^31^, Parsi and Bairamov^32^ and Metropolis et al.^33^ are just a few of the studies that worked with the MCMC technique. From Eq. (15) the conditional posterior density function of $\alpha _{1},\ \alpha _{2}~, \beta _{1}$ and $\ \beta _{2}$ can be obtained as the following proportionality to simplify, we used $\pi ^{*}_{1}\left( \alpha _{1}\right) , ~\pi ^{*}_{2}\left( \alpha _{2}\right) , ~\pi ^{*}_{3}\left( \beta _{1} \right)$ and $\pi ^{*}_{4}\left( \beta _{2} \right)$ instead of $\pi ^{*}_{1}\left( \alpha _{1}\mid \alpha _{2},\beta _{1}, \beta _{2}, H, \lambda , S\right) , ~\pi ^{*}_{2}\left( \alpha _{2}\mid \alpha _{1},\beta _{1}, \beta _{2}, H, \lambda , S\right) , ~\pi ^{*}_{3}\left( \beta _{1}\mid \alpha _{1},\alpha _{2}, \beta _{2}, H, \lambda , S \right)$ and $\pi ^{*}_{4}\left( \beta _{2}\mid \alpha _{1},\alpha _{2},\beta _{1}, H, \lambda , S \right)$ respectively:

16 $$\begin{aligned}{} & {} \pi _{1}^{*}\left( \alpha _{1}\right) \propto \alpha _{1}^{-2m_{r}+a_{1}-1}e^{-b_{1} \alpha _{1}} e^{-\sum _{i=1}^{r}\frac{h_{i}\lambda _{i}^{2\beta _{1}}}{2\alpha _{1}^{2}}}e^{-\sum _{i=1}^{r}\frac{s_{i}\lambda _{i}^{2\beta _{1}}}{\alpha _{1}^{2}}}, \end{aligned}$$

17 $$\begin{aligned}{} & {} \pi _{2}^{*}\left( \alpha _{2}\right) \propto \alpha _{2}^{-2n_{r}+a_{2}-1}e^{-b_{2} \alpha _{2}} e^{-\sum _{i=1}^{r}\frac{(1-h_{i})\lambda _{i}^{2\beta _{2}}}{\alpha _{1}^{2}}}e^{-\sum _{i=1}^{r}\frac{t_{i}\lambda _{i}^{2\beta _{2}}}{\alpha _{2}^{2}}}, \end{aligned}$$

18 $$\begin{aligned}{} & {} \pi _{3}^{*}\left( \beta _{1}\right) \propto \beta _{1}^{m_{r}+a_{3}-1}e^{-b_{3} \beta _{1}} \prod \limits _{i=1}^{r}\lambda _{i}^{(2\beta _{1}-1)h_{i}} e^{-\sum _{i=1}^{r}\frac{h_{i}\lambda _{i}^{2\beta _{1}}}{2\alpha _{1}^{2}}} e^{-\sum _{i=1}^{r}\frac{s_{i} \lambda _{i}^{2\beta _{1}}}{\alpha _{1}^{2}}}, \end{aligned}$$

and

19 $$\begin{aligned} \pi _{4}^{*}\left( \beta _{2}\right) \propto \beta _{2}^{n_{r}+a_{4}-1}e^{-b_{4} \beta _{2}} \prod \limits _{i=1}^{r}\lambda _{i}^{(2\beta _{2}-1) (1-h_{i})}e^{-\sum _{i=1}^{r}\frac{(1-h_{i})\lambda _{i}^{2\beta _{2}}}{2\alpha _{2}^{2}}} e^{-\sum _{i=1}^{r} \frac{t_{i}\lambda _{i}^{2\beta _{2}}}{\alpha _{2}^{2}}}. \end{aligned}$$

**Figure 3:**
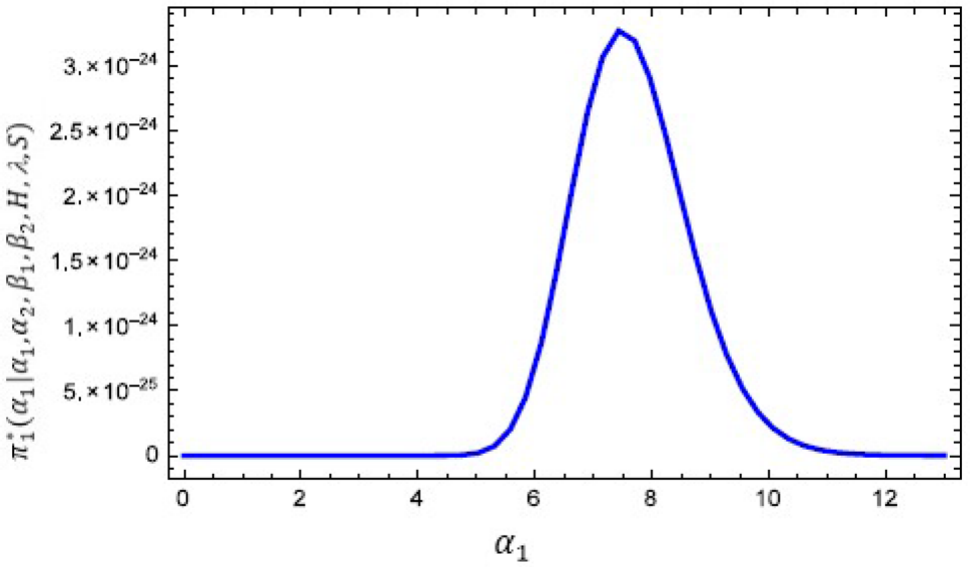
The conditional posterior density of MCMC results of the $\alpha _{1}$ parameter.

**Figure 4:**
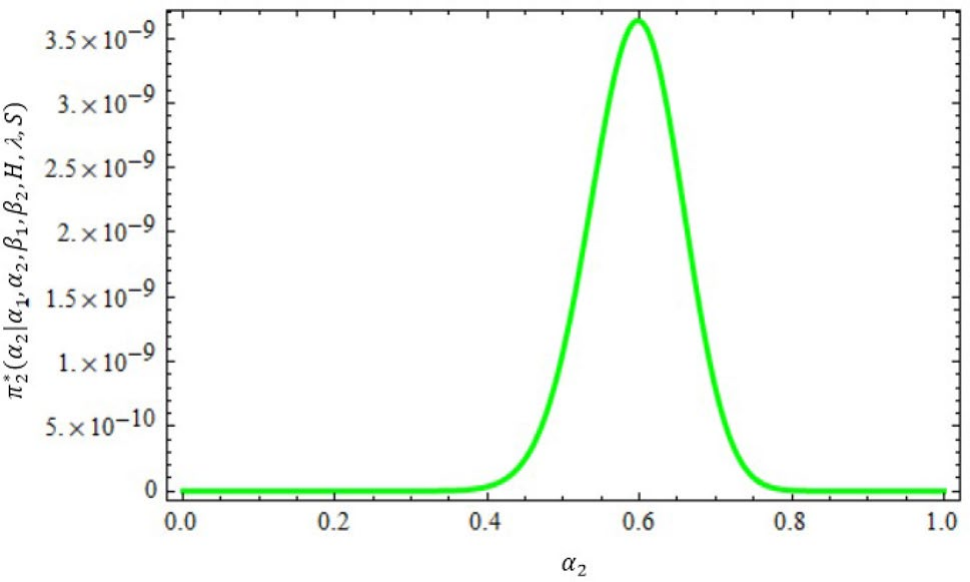
The conditional posterior density of MCMC results of the $\alpha _{2}$ parameter.

**Figure 5:**
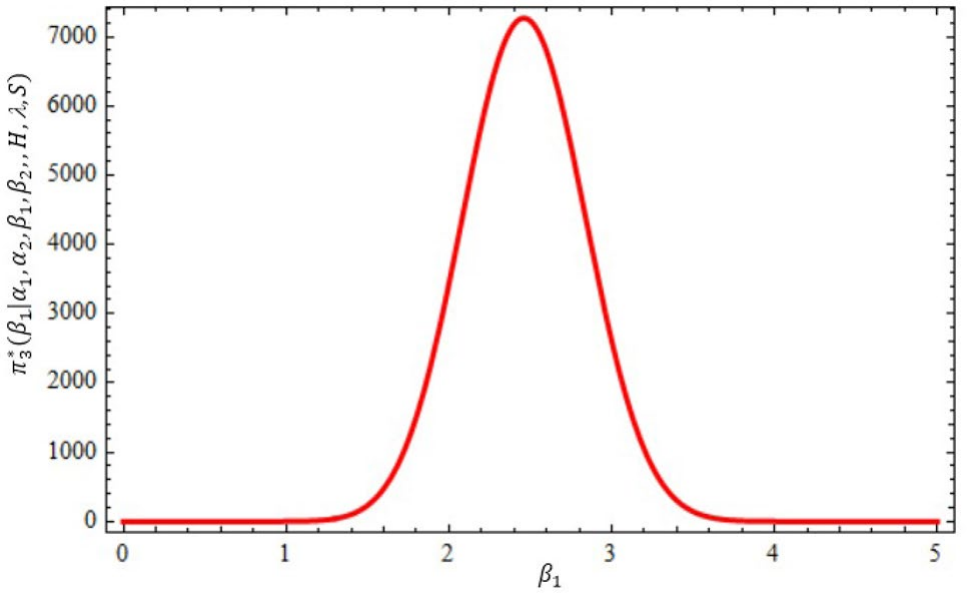
The conditional posterior density of MCMC results of the $\beta _{1}$ parameter.

**Figure 6:**
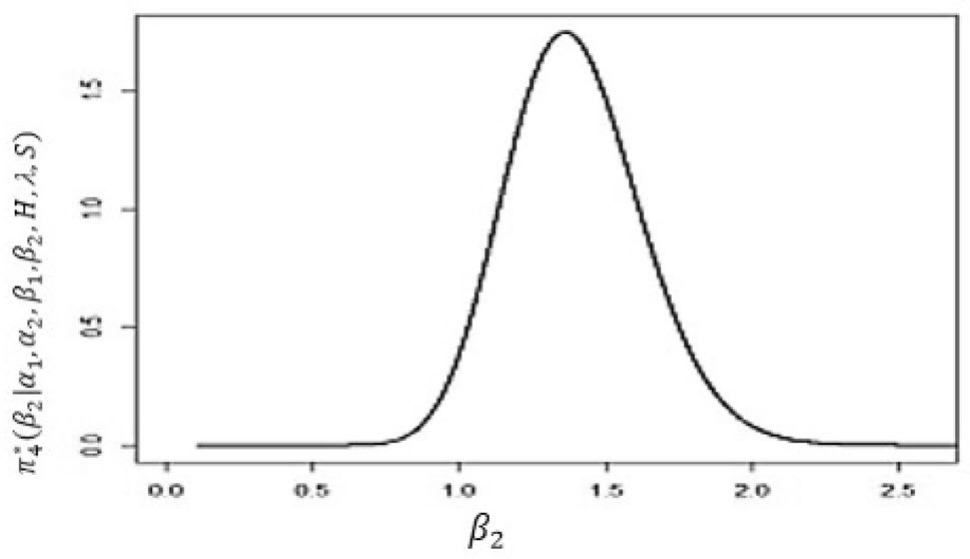
The conditional posterior density of MCMC results of the $\beta _{2}$ parameter.

The conditional posterior function of $\alpha _{1},\ \alpha _{2}~, \beta _{1}$ and $\ \beta _{2}$ in Eqs. (16)–(19) cannot be reduced analytically to well known distributions. Consequently, it is difficult sample directly by standard methods, but the plot of them see in Figs. 3, 4, 5 and 6 display that they are similar to normal distribution.

### Gibbs sampling

To produce the Bayesian estimate of unknown parameters and the related credible interval, we now employ the Gibbs sampling method, which is a subclass of Markov chain Monte-Carlo (MCMC) methods. Using the posterior conditional density functions of the parameters $\alpha _{1},\ \alpha _{2}~$, $\ \beta _{1}$ and $\ \beta _{2}$, this approach produces posterior samples. Eq. (15) identifies the posterior density function of the parameters $\alpha _{1},\ \alpha _{2}~$, $\ \beta _{1}$ and $\ \beta _{2}$. As indicated by Eqs. (16)–(19), the conditional density function of $\alpha _{1},\ \alpha _{2}~$, $\ \beta _{1}$ and $\ \beta _{2}$ cannot be achieved in the form of the well-known density functions (19). As a result, we use the^33^, Metropolis–Hasting (MH) algorithm uses a normal proposal distribution to generate random samples from the posterior densities of $\alpha _{1},\ \alpha _{2}~$, $\ \beta _{1}$ and $\ \beta _{2}$.

The steps of Gibbs sampling are described as follows:

- *Step 1*. Start with an initial guess $(\alpha _{1}^{(0)},\alpha _{2}^{(0)},\beta _{1}^{(0)},\beta _{2}^{(0)})$ =$(\hat{\alpha _{1}},\hat{\alpha _{2}},\hat{\beta _{1}},\hat{\beta _{2}})$.
- *Step 2*. Set $$t = 1$$.
- *Step 3*. Generate $(\alpha _{1}^{(t)},\alpha _{2}^{(t)},\beta _{1}^{(t)}, \beta _{2}^{(t)})$ from $\pi _{1}^{*}(\alpha _{1}^{(t-1)}\mid H, \lambda , S)$, $\pi _{2}^{*}(\alpha _{2}^{(t-1)}\mid H, \lambda , S)$, $\pi _{3}^{*}(\beta _{1}^{(t-1)}\mid H, \lambda , S)$, and $\pi _{4}^{*}(\beta _{2}^{(t-1)}\mid H, \lambda , S)$ using MH algorithm with the proposal distributions $N(\alpha _{1}^{(t-1)}, \sqrt{\widehat{var(\alpha _{1})}})$, $N(\alpha _{2}^{(t-1)}, \sqrt{\widehat{var(\alpha _{2})}})$, $N(\beta _{1}^{(t-1)}, \sqrt{\widehat{var(\beta _{1})}})$,  and $N(\beta _{2}^{(t-1)}, \sqrt{\widehat{var(\beta _{2})}})$ respectively.
  (i) Generate proposals $\alpha _{1}^{*}$ from $N(\alpha _{1}^{(t-1)}, \sqrt{\widehat{var(\alpha _{1})}})$, $\alpha _{2}^{*}$ from $N(\alpha _{2}^{(t-1)}, \sqrt{\widehat{var(\alpha _{2})}})$, $\beta _{1}^{*}$ from $N(\beta _{1}^{(t-1)}, \sqrt{\widehat{var(\beta _{1})}})$, and $\beta _{2}^{*}$ from $N(\beta _{2}^{(t-1)}, \sqrt{\widehat{var(\beta _{2})}})$.
  (ii) Measure the acceptance probabilities $\eta _{\alpha _{1}}=\min \left( 1, \frac{\pi _{1}^{*}(\alpha _{1}^{(t)}\mid H, \lambda , S)}{\pi _{1}^{*}(\alpha _{1}^{(t-1)}\mid H, \lambda , S)}\right)$, $\eta _{\alpha _{2}}=\min \left( 1, \frac{\pi _{2}^{*}(\alpha _{2}^{(t)}\mid H, \lambda , S)}{\pi _{2}^{*}(\alpha _{2}^{(t-1)}\mid H, \lambda , S)}\right)$, $\eta _{\beta _{1}}=\min \left( 1, \frac{\pi _{3}^{*}(\beta _{1}^{(t)}\mid H, \lambda , S)}{\pi _{3}^{*}(\beta _{1}^{(t-1)}\mid H, \lambda , S)}\right)$,  and $\eta _{\beta _{2}}=\min \left( 1, \frac{\pi _{4}^{*}(\beta _{2}^{(t)}\mid H, \lambda , S)}{\pi _{4}^{*}(\beta _{2}^{(t-1)}\mid H, \lambda , S)}\right)$.
  (iii) Generate $u_{1}$, $u_{2}$, $u_{3}$ and $u_{4}$ from Uniform (0, 1).
  (iv) If $u_{1} < \eta _{\alpha _{1}}$, accept the proposal and set $(\alpha _{1}^{(t)})=(\alpha _{1}^{(*)})$ else, set $(\alpha _{1}^{(t)})=(\alpha _{1}^{(t-1)})$.
  (v) If $u_{2} < \eta _{\alpha _{2}}$, accept the proposal and set $(\alpha _{2}^{(t)})=(\alpha _{2}^{(*)})$ else, set $(\alpha _{2}^{(t)})=(\alpha _{2}^{(t-1)})$.
  (vi) If $u_{3} < \eta _{\beta _{1}}$, accept the proposal and set $(\beta _{1}^{(t)})=(\beta _{1}^{(*)})$ else, set $(\beta _{1}^{(t)})=(\beta _{1}^{(t-1)})$.
  (vii) If $u_{4} < \eta _{\beta _{2}}$, accept the proposal and set $(\beta _{2}^{(t)})=(\beta _{2}^{(*)})$ else, set $(\beta _{2}^{(t)})=(\beta _{2}^{(t-1)})$.
- *Step 4*. Set $$t = t + 1$$.
- *Step 5*. Repeat Steps (3)–(5) *N* times and get the posterior sample to estimate the unknown parameters $\alpha _{1},\ \alpha _{2}~, \beta _{1}$ and $\ \beta _{2}$.

## Application of real data

In this section, we analyse a data set primarily for illustration purposes. Rasouli and Balakrishnan^6^ also used these data sets, originally obtained from^34^. The data includes the time intervals (in hours) between air conditioning system failures on a fleet of 13 Boeing 720 jet planes. For illustration purposes, we used the planes “7913” and “7914”. The following data is provided:

- PLANE 7914: 3, 5, 5, 13, 14, 15, 22, 22, 23, 30, 36, 39, 44, 46, 50, 72, 79, 88, 97, 102, 139, 188, 197, 210.
- PLANE 7913: 1, 4, 11, 16, 18, 18, 18, 24, 31, 39, 46, 51, 54, 63, 68, 77, 80, 82, 97, 106, 111, 141, 142, 163,  191, 206, 216.

For each sample, we fit the Power Rayleigh distribution and provide the results in Table 3. The Kolmogorov–Smirnov test statistic values (K–S) and corresponding *p* values were provided, indicating that the data fit the Power Rayleigh distribution with the parameters presented in Table 1.

**Table 1:** MLEs and Kolmogorov–Smirnov test results for data.

Data	$\hat{\alpha }_{i}$	$\hat{\beta }_{i}$	K–S	*P* value
X	0.0277872	0.512578	0.0891497	0.98203
Y	0.0145383	0.561924	0.0885429	0.971488

**Figure 7:**
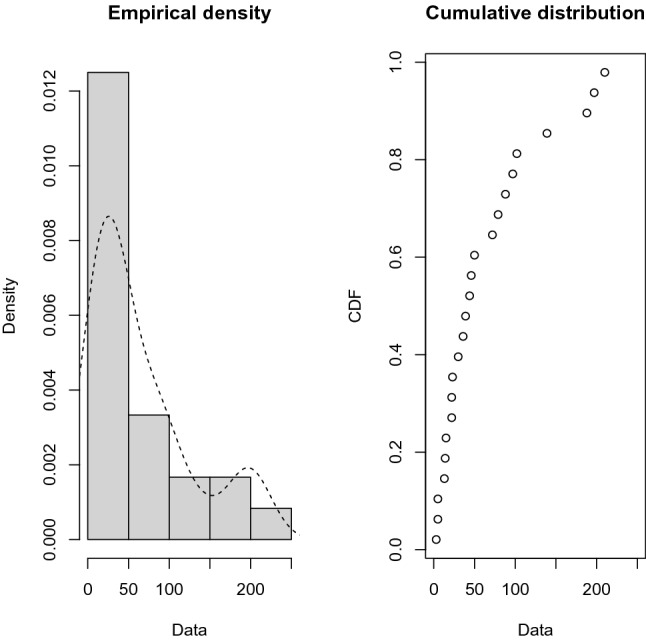
The Empirical density and cumulative distribution for the first sample of real data.

**Figure 8:**
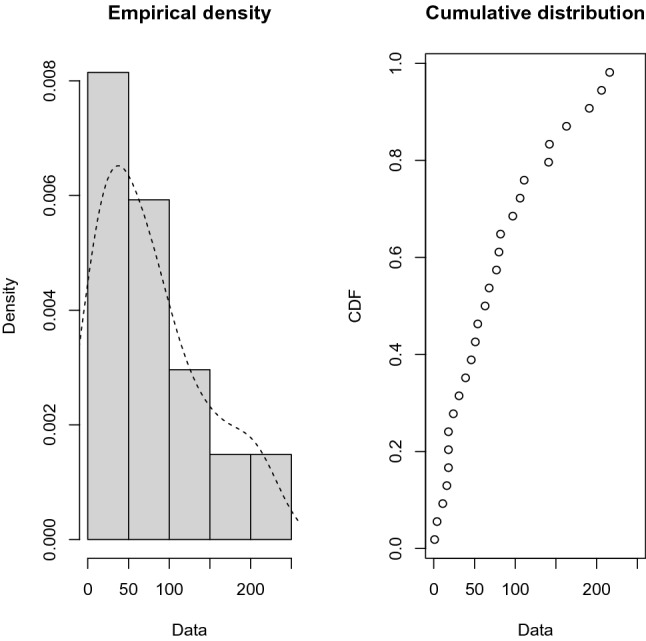
The Empirical density and cumulative distribution for the second sample of real data.

So, the power Rayleigh distribution fits the data very well in both samples, and we have just plotted the empirical and fitted it in Fig. 7 for the first sample and Fig. 8 for the second sample. It is evident that the power Rayleigh distribution can be a better model for fitting this data. From the above data sets, we have generated JP-II-C sample with the censoring scheme. Assume that $m = 24$ for the first sample and $n = 27$ for the second sample, by implementing JP-II-CS where $K = m + n$ denotes the total sample size, and when $r = 10, S = ( 5, 0, 0, 0, 5, 0, 0, 0, 0, 8)$, $T = (5, 0, 0, 0, 5, 0, 0, 0, 0, 10),$ and $R = (10, 0, 0, 0, 10, 0, 0, 0, 0, 18)$. The generated data sets are provided below

$$\begin{aligned} \lambda = (2.2, 3.3, 3.4, 3.4, 3.5, 3.6, 3.7, 3.8, 3.8, 3.8), \end{aligned}$$

and

$$\begin{aligned} H = (1, 0, 1, 1, 0, 1, 0, 1, 1, 1). \end{aligned}$$

**Figure 9:**
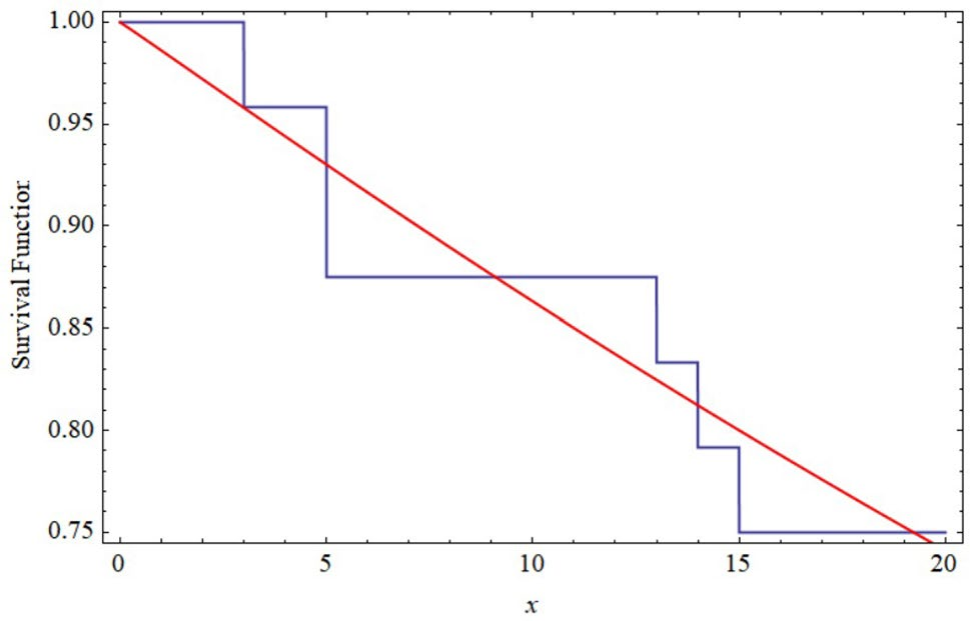
The Empirical and fitted survival functions for the first sample of real data.

**Figure 10:**
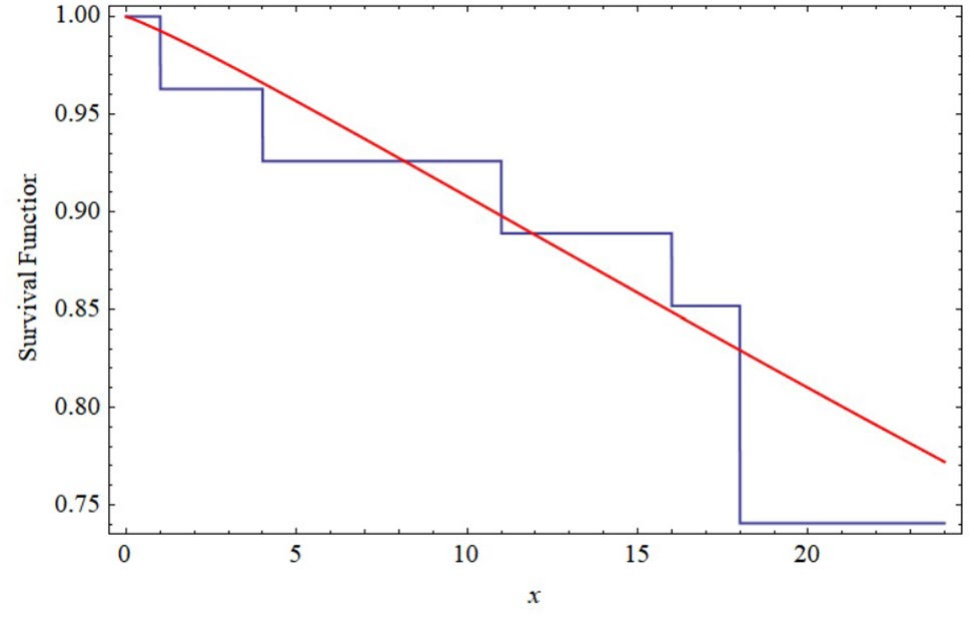
The Empirical and fitted survival functions for the second sample of real data.

Based on the above JP-II-CS sample, we compute the point estimate based on MLEs and the results of $95\%$ ACIs for $\alpha _{1},\alpha _{2},\beta _{1}~$, and $\beta _{2}$, the results of which are shown in Tables 2 and 3. For Bayesian estimation, we used MCMC method based on 10, 000 MCMC sample and discard the first 1000 values as ‘burn-in’. We used the informative priors which follow the Gamma distribution with hyperparameters $a_{i}=0.02$ and $b_{i}=2$. Table 2 shows the Bayesian estimates for $\alpha _1, \alpha _2, \beta _1$, and $\beta _2$ under the SE and LINEX loss functions. The two samples can be seen that the power Rayleigh distribution fits the data very well and also we have just plotted the empirical S(t) and the fitted S(t) in Fig. 9 for the first sample and in Fig. 10 for the second sample. It is evident that the power Rayleigh distribution can be a good model fitting this data. Moreover, the results of the $95\%$ CRIs for $\alpha _{1},\alpha _{2},\beta _{1}~$ and $\beta _{2}~$ are tabled in Table 3. As we can see, the variances of $\alpha _{1},\alpha _{2},$ and $\beta _{1}~$ are very large comparing to their values. This would lead to a negative lower bound of the asymptotic confidence intervals of $\alpha _{1},\alpha _{2},$ and $\beta _{1}~$. Since $\alpha _{1},\alpha _{2},$ and $\beta _{1}~$ cannot be negative, we truncate the lower limits at zero. This is one of the disadvantages of the maximum likelihood method.

**Table 2:** Different point estimates for the parameters $\alpha _{1},\alpha _{2},\beta _{1}\text { and }\beta _{2}$.

Parameters	MLE	SEL	LINEX		
			$c=-2$	$c=0.0001$	$c=2$
$\alpha _{1}$	0.9785	0.9782	0.9782	0.9782	0.9582
$\alpha _{2}$	1.363	1.3635	1.3635	1.3635	1.3624
$\beta _{1}$	1.5671	1.5677	1.5677	1.5677	1.5247
$\beta _{2}$	1.8011	1.8014	1.8014	1.8014	1.7014

**Table 3:** $$95\%$$ $$\text {CIs of }\alpha _{1},\alpha _{2},\beta _{1} \text { and }\beta _{2}.$$.

Parameter	MLE	Length	MCMC	Length
$\alpha _{1}$	(0, 2.889)	0.7279	(0.599, 1.851)	0.0037
$\alpha _{2}$	(0, 3.377)	0.6779	(0.7828, 2.993)	0.0087
$\beta _{1}$	(0, 2.762)	2.5304	(0.313, 1.987)	0.0076
$\beta _{2}$	(0.616, 1.634)	2.8918	(0.791, 2.217)	0.0074

**Figure 11:**
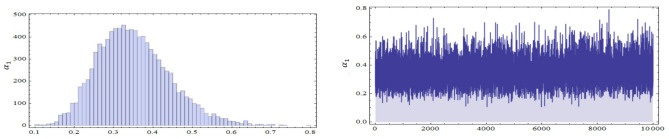
The posterior density function and the trace plots for the parameters $\alpha _{1}$.

**Figure 12:**
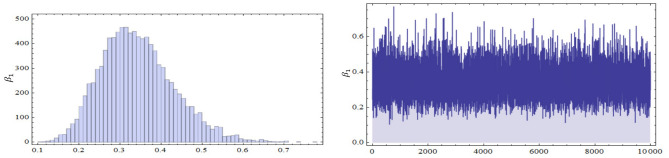
The posterior density function and the trace plots for the parameters $\beta _{1}$.

**Figure 13:**
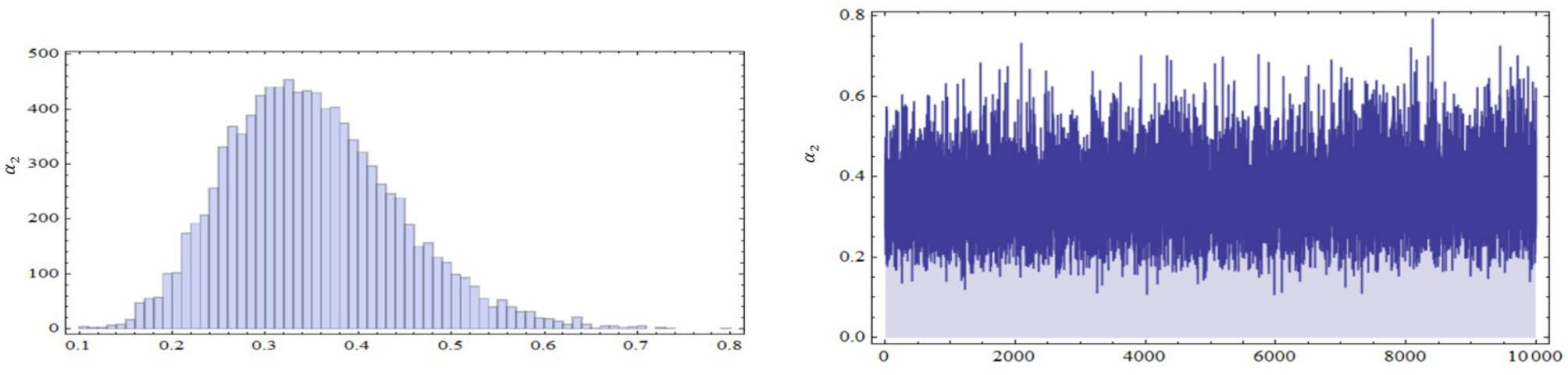
The posterior density function and the trace plots for the parameters $\alpha _{2}$.

**Figure 14:**
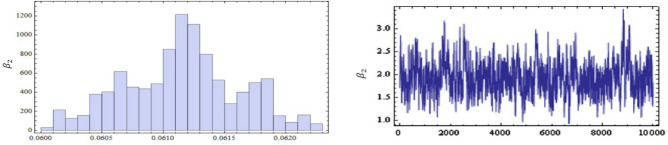
The posterior density function and the trace plots for the parameters $\beta _{2}$.

Table 4 shows the comparison between the approximation of the expected values of the number of failures from the first production line before the test performance (A.E.B) and the mean of the exact number of failures after the test performance (M.E.A) when $r=15,20,25,30$ and 35. The plots of the posterior density functions and the trace plots of the unknown parameters $\alpha _{1}$, $\alpha _{2}$, $\beta _{1}$, and $\beta _{2}$ using MCMC method have been shown in Figs. 11, 12, 13 and 14.

**Table 4:** The comparison between A.E.B and A.E.A for real data.

Scheme no.	$\left( n,m\right)$	*r*	($R_{1,.....,}R_{r}$)	*p* = 0.28		*p* = 0.72	
				A.E.B	A.E.A	A.E.B	A.E.A
1	$\left( 27,24\right)$	5	(15, 0, 0, 0, 0)	6.1758	9.1854	6.758	9.154
2		15	(0, 0, 10, 5, 0)	8.2664	12.9661	8.664	12.5661
3		20	(0, 0, 0, 0, 15)	11.698	14.8549	11.998	14.2549
4		25	$(10,0_{(9)})$	13.478	16.7515	13.898	16.115
5		30	$(0_{(5)},10,0_{(4)})$	15.975	17.0708	16.75	17.018

## Simulation

A simulation study was performed to compare the performance of the different methods discussed in this paper. Suppose various sample sizes for the two populations as $m, n = 10,20,30$, and various values of $r = 5,10,15,20,30,40$. Also, set the parameters $(\alpha _{1}^{(t)},\alpha _{2}^{(t)},\beta _{1}^{(t)}, \beta _{2}^{(t)}) = (0.5,0.6,2.5,2.69,0.69,0.8,1.57,1.8)$. The MSEs, lengths of 95 % coverage probability (CP) for the parameters $(\alpha _{1}^{(t)},\alpha _{2}^{(t)},\beta _{1}^{(t)}$, and $\beta _{2}^{(t)})$ have been evaluated using MLEs and MCMC with 10000 observations under SE and LINEX loss functions. This process is repeated 1000 times and the results of the mean values of MSE, lengths and CP, are displayed in Tables 5, 6, 7, and 8. Moreover, in this section a simulation study was conducted to compute the expected number of failures from the first production line (S.E.Mr) and also compute the approximated expected number of failures (A.E.Mr). We assumed various sample sizes for the two populations as$~ m, n = 5,10,15, 20,25,30,40;50$ and various choices of JP-II-CS $r = 5,10,20,30,40$ samples from the two PRD populations have been generated under the same truth values of these parameters, the results are presented in Table 9. The calculations in Table 9 are computed under the following assumptions: $p = P(X_1 < X_2)$ where $X_1, X_2$ are the lifetime of the first production line units and the second production line units, respectively, in which $X_1$ is selected from PRD(0.5, 2.5),  and $X_2$ from PRD(0.6, 2.69), once more $X_1$ selected from PRD(0.8, 1.8),  and $X_2$ from PRD(0.69, 1.57). We calculate the A.E.Mr according to Parsi and Bairamov^32^ as follows:

**Table 5:** MSE, length and coverage probability (CP) of estimates for the parameter$\alpha _{1}$.

$\left( n,m\right)$	*r*	($R_{1,.....,}R_{r}$)	MLE			SEL	LINEX			Length	CP
			MSE	Length	CP		$\ \ c=-2$	$c=0.0001$	$\ \ \ c=2$		
$\left( 10,10\right)$	5	(15, 0, 0, 0, 0)	0.1758	1.1854	0.9592	0.1758	0.1758	0.1758	0.0063	0.0063	0.9502
		(0, 0, 10, 5, 0)	0.2664	1.9661	0.9671	0.267	0.267	0.267	0.121	0.0092	0.9642
		(0, 0, 0, 0, 15)	0.221	1.8549	0.9327	0.2222	0.2222	0.2222	0.213	0.0086	0.9430
	10	$(10,0_{(9)})$	0.0401	0.7515	0.9713	0.0401	0.0401	0.0401	0.011	0.0047	0.9355
		$(0_{(5)},10,0_{(4)})$	0.2435	1.0708	0.9559	0.2431	0.2431	0.2431	0.144	0.0047	0.9306
		$(0_{(9)},10)$	0.1554	0.9049	0.9470	0.1553	0.1553	0.1553	0.1055	0.0041	0.9391
$\left( 10,20\right)$	15	(15,0$_{(14)})$	0.1909	0.8929	0.9684	0.1908	0.1908	0.1908	0.1708	0.0042	0.9319
		(0$_{(6)},15,0_{(8)}$)	0.1907	0.951	0.9428	0.1899	0.1899	0.1899	0.1589	0.0049	0.9265
		(0$_{(14)},15$)	0.2092	0.9846	0.9633	0.2089	0.2089	0.2089	0.1089	0.0045	0.9689
$\left( 10,20\right)$	20	(10,0$_{(19)})$	0.2325	0.8856	0.9747	0.2324	0.2324	0.2324	0.1324	0.0038	0.9581
		(0$_{(9)},10,0_{(10)}$)	0.2422	0.9387	0.9470	0.2415	0.2415	0.2415	0.2241	0.004	0.9552
		(0$_{(19)},10$)	0.3419	0.993	0.9536	0.3412	0.3412	0.3412	0.3012	0.004	0.9652
(20,30)	20	(30,0$_{(19)}$)	0.1799	0.7279	0.9527	0.1799	0.1799	0.1799	0.1579	0.0037	0.9320
		(0$_{(9)},30,$0$_{(10)}$)	0.1715	0.7251	0.9266	0.171	0.171	0.171	0.156	0.0036	0.9475
		(0$_{(19)},30)$	0.2313	0.8595	0.9443	0.2307	0.2307	0.2307	0.2105	0.004	0.9737
(20,30)	30	(20,0$_{(29)}$)	0.1996	0.6248	0.9529	0.1995	0.1995	0.1995	0.1785	0.0029	0.9420
		(0$_{(19)},20,$0$_{(10)}$)	0.1927	0.5973	0.9698	0.1931	0.1931	0.1931	0.1832	0.0027	0.9677
		(0$_{(29)},20)$	0.2358	0.6338	0.9433	0.2359	0.2359	0.2359	0.2148	0.0027	0.9668
(30,20)	30	(20,0$_{(29)}$)	0.1804	0.4823	0.9616	0.1804	0.1804	0.1804	0.1503	0.0022	0.93516
		(0$_{(19)},20,$0$_{(10)}$)	0.1468	0.4403	0.9563	0.147	0.147	0.147	0.132	0.0022	0.9683
		(0$_{(29)},20)$	0.1193	0.4259	0.9520	0.1193	0.1193	0.1193	0.1134	0.0021	0.9364
(30,20)	40	(10,0$_{(39)})$	0.2047	0.4454	0.9380	0.2046	0.2046	0.2046	0.1948	0.002	0.9269
		(0$_{(19)},10,0_{(20)}$)	0.1837	0.4154	0.9269	0.1835	0.1835	0.1835	0.1354	0.0018	0.9593
		(0$_{(39)},10$)	0.1694	0.3984	0.9650	0.1694	0.1694	0.1694	0.1253	0.0019	0.9354

**Table 6:** MSE, length and coverage probability (CP) of estimates for the parameter$\text { }\alpha _{2}$.

$\left( n,m\right)$	*r*	($R_{1,.....,}R_{r}$)	MLE			SEL	LINEX			Length	CP
			MSE	Length	CP		$\ \ c=-2$	c=0.0001	$\ \ \ c=2$		
$\left( 10,10\right)$	5	(15,0,0,0,0)	0.352	1.1854	0.9518	0.3526	0.3526	0.3526	0.0087	0.0063	0.9278
		(0,0,10,5,0)	0.2831	2.7316	0.9507	0.2819	0.2819	0.2819	0.0106	0.0092	0.9524
		(0,0,0,0,15)	0.1989	3.4005	0.9579	0.2002	0.2002	0.2002	0.140	0.0145	0.9263
	10	$(10,0_{(9)})$	0.0556	0.866	0.9611	0.0558	0.0558	0.0558	0.011	0.0056	0.9356
		$(0_{(5)},10,0_{(4)})$	0.4111	1.9832	0.9735	0.4119	0.4119	0.4119	0.321	0.0066	0.9279
		$(0_{(9)},10)$	0.177	1.7028	0.9270	0.1772	0.1772	0.1772	0.1572	0.0069	0.9302
$\left( 10,20\right)$	15	(15,0$_{(14)})$	0.3623	1.3678	0.9300	0.3614	0.3615	0.3614	0.2614	0.0045	0.9347
		(0$_{(6)},15,0_{(8)}$)	0.345	1.2532	0.9631	0.3452	0.3452	0.3452	0.2451	0.0043	0.9488
		(0$_{(14)},15$)	0.137	0.9855	0.9639	0.1368	0.1368	0.1368	0.1357	0.004	0.9712
$\left( 10,20\right)$	20	(10,0$_{(19)})$	0.3759	1.2143	0.9672	0.3749	0.3749	0.3749	0.2649	0.0041	0.9329
		(0$_{(9)},10,0_{(10)}$)	0.2669	0.9893	0.9293	0.2672	0.2672	0.2672	0.2532	0.0036	0.9749
		(0$_{(19)},10$)	0.1099	0.7222	0.9609	0.1103	0.1103	0.1103	0.1003	0.0033	0.9584
(20,30)	20	(30,0$_{(19)}$)	0.3617	1.2457	0.9638	0.3625	0.3625	0.3625	0.2862	0.0041	0.9451
		(0$_{(9)},30,$0$_{(10)}$)	0.263	1.0633	0.9569	0.2625	0.2625	0.2625	0.1825	0.0035	0.9435
		(0$_{(19)},30)$	0.2024	1.3269	0.9457	0.2023	0.2023	0.2023	0.1023	0.005	0.9472
(20,30)	30	(20,0$_{(29)}$)	0.3468	0.9906	0.9607	0.3461	0.3461	0.3461	0.2546	0.0034	0.9516
		(0$_{(19)},20,$0$_{(10)}$)	0.2564	0.8448	0.9396	0.2562	0.2562	0.2562	0.2312	0.0029	0.99288
		(0$_{(29)},20)$	0.0981	0.6504	0.9460	0.0982	0.0982	0.0982	0.0781	0.0026	0.9559
(30,20)	30	(20,0$_{(29)}$)	0.3636	1.2674	0.9287	0.3646	0.3646	0.3646	0.2462	0.0043	0.9661
		(0$_{(19)},20,$0$_{(10)}$)	0.2769	1.0825	0.9693	0.2768	0.2768	0.2768	0.1769	0.0039	0.9346
		(0$_{(29)},20)$	0.1518	0.9669	0.99283	0.1514	0.1514	0.1514	0.1124	0.004	0.9740
(30,20)	40	(10,0$_{(39)})$	0.3936	1.1379	0.9581	0.3936	0.3936	0.3936	0.3365	0.0037	0.9671
		(0$_{(19)},10,0_{(20)}$)	0.3144	0.9896	0.9491	0.3144	0.3144	0.3144	0.2914	0.0034	0.9305
		(0$_{(39)},10$)	0.1938	0.8075	0.9278	0.1938	0.1938	0.1938	0.1089	0.0029	0.9670

**Table 7:** MSE, length and coverage probability (CP) of estimates for the parameter$\text { }\beta _{1}$.

$\left( n,m\right)$	*r*	($R_{1,.....,}R_{r}$)	MLE			SEL	LINEX			Length	CP
			MSE	Length	CP		$\ \ c=-2$	c=0.0001	$\ \ \ c=2$		
$\left( 10,10\right)$	5	(15,0,0,0,0)	0.1236	4.1966	0.9370	0.123	0.123	0.123	0.012	0.012	0.9458
		(0,0,10,5,0)	0.1123	5.2603	0.9553	0.1134	0.1134	0.1133	0.023	0.0153	0.9684
		(0,0,0,0,15)	0.1178	5.5392	0.9364	0.1177	0.1177	0.1177	0.011	0.0156	0.9382
	10	$(10,0_{(9)})$	0.1261	4.6473	0.9679	0.1243	0.1243	0.1243	0.023	0.0139	0.9525
		$(0_{(5)},10,0_{(4)})$	0.1005	3.3984	0.9413	0.1006	0.1006	0.1006	0.061	0.0099	0.9553
		$(0_{(9)},10)$	0.113	3.774	0.9419	0.1129	0.1129	0.1129	0.0229	0.0104	0.9580
$\left( 10,20\right)$	15	(15,0$_{(14)})$	0.1025	3.5665	0.9386	0.1022	0.1022	0.1022	0.0122	0.0101	0.9440
		(0$_{(6)},15,0_{(8)}$)	0.1123	3.4124	0.9419	0.1124	0.1124	0.1124	0.1104	0.0102	0.9258
		(0$_{(14)},15$)	0.0997	3.4153	0.9348	0.1014	0.1014	0.1014	0.1002	0.0106	0.9625
$\left( 10,20\right)$	20	(10,0$_{(19)})$	0.0929	2.8418	0.9277	0.0927	0.0927	0.0927	0.0827	0.0085	0.9350
		(0$_{(9)},10,0_{(10)}$)	0.0916	3.02	0.9251	0.0915	0.0915	0.0915	0.0903	0.0087	0.9622
		(0$_{(19)},10$)	0.1043	2.8814	0.9623	0.1041	0.1041	0.1041	0.1021	0.0086	0.9392
(20,30)	20	(30,0$_{(19)}$)	0.0748	2.5304	0.9253	0.0742	0.0742	0.0742	0.0554	0.0076	0.9345
		(0$_{(9)},30,$0$_{(10)}$)	0.1021	2.6785	0.9659	0.1021	0.1021	0.1021	0.0521	0.0075	0.9402
		(0$_{(19)},30)$	0.0886	2.7242	0.9291	0.0875	0.0875	0.0875	0.0475	0.0085	0.9645
(20,30)	30	(20,0$_{(29)}$)	0.0865	2.1976	0.99519	0.0829	0.0829	0.0829	0.0326	0.0065	0.9575
		(0$_{(19)},20,$0$_{(10)}$)	0.0875	2.1912	0.9255	0.0867	0.0867	0.0867	0.0356	0.0062	0.9603
		(0$_{(29)},20)$	0.0853	2.2686	0.9338	0.0852	0.0852	0.0852	0.0286	00068	0.9565
(30,20)	30	(20,0$_{(29)}$)	0.0575	1.7424	0.9485	0.0578	0.0578	0.0578	0.0437	0.005	0.9440
		(0$_{(19)},20,$0$_{(10)}$)	0.0581	1.6021	0.9472	0.0581	0.0581	0.0581	0.0574	0.0051	0.9405
		(0$_{(29)},20)$	0.091	2.0767	0.9668	0.0911	0.0911	0.0821	0.0861	0.0057	0.9351
(30,20)	40	(10,0$_{(39)})$	0.0516	1.5204	0.9414	0.0516	0.0516	0.0516	0.0425	0.0045	0.9564
		(0$_{(19)},10,0_{(20)}$)	0.0577	1.48	0.9743	0.0576	0.0576	0.0576	0.0479	0.0043	0.9256
		(0$_{(39)},10$)	0.0618	1.6109	0.9659	0.0622	0.0622	0.0622	0.0534	0.0047	0.9423

**Table 8:** MSE, length and coverage probability (CP) of estimates for the parameter$\text { }\beta _{2}$.

$\left( n,m\right)$	*r*		MLE			SEL	LINEX			Length	CP
		($R_{1,.....,}R_{r}$)	MSE	Length	CP		$\ \ c=-2$	c=0.0001	$\ \ \ c=2$		
$\left( 10,10\right)$	5	(15,0,0,0,0)	0.0875	5.504	0.9322	0.0878	0.0878	0.0878	0.0141	0.014	0.9312
		(0,0,10,5,0)	0.1093	6.8421	0.9377	0.1099	0.1099	0.1099	0.0123	0.0188	0.9574
		(0,0,0,0,15)	0.0902	8.8252	0.9321	0.0895	0.0895	0.0895	0.056	0.0236	0.9272
	10	$(10,0_{(9)})$	0.0716	6.3171	0.9462	0.0705	0.0705	0.0705	0.0454	0.0165	0.9418
		$(0_{(5)},10,0_{(4)})$	0.0702	4.5537	0.9720	0.0704	0.0704	0.0704	0.0431	0.0118	0.9437
		$(0_{(9)},10)$	0.1299	5.738	0.9576	0.1304	0.1304	0.1304	0.1204	0.0162	0.9704
$\left( 10,20\right)$	15	(15,0$_{(14)})$	0.0732	3.1685	0.9386	0.0736	0.0736	0.0736	0.0368	0.0088	0.9440
		(0$_{(6)},15,0_{(8)}$)	0.0837	3.0532	0.9419	0.0843	0.0843	0.0843	0.0832	0.008	0.9258
		(0$_{(14)},15$)	0.0819	3.8731	0.9348	0.0824	0.0824	0.0824	0.0813	0.0104	0.9625
$\left( 10,20\right)$	20	(10,0$_{(19)})$	0.0602	2.6966	0.9277	0.0599	0.0599	0.0599	0.0489	0.0067	0.9251
		(0$_{(9)},10,0_{(10)}$)	0.0744	2.7524	0.9622	0.0744	0.0744	0.0744	0.0724	0.007	0.9623
		(0$_{(19)},10$)	0.0619	3.4295	0.9392	0.0623	0.0623	0.0623	0.0513	0.009	0.9253
(20,30)	20	(30,0$_{(19)}$)	0.0775	2.8918	0.9345	0.0775	0.0775	0.0775	0.0675	0.0074	0.9659
		(0$_{(9)},30,$0$_{(10)}$)	0.0923	2.5505	0.9402	0.0927	0.0927	0.0927	0.0537	0.0068	0.9291
		(0$_{(19)},30)$	0.0877	3.8966	0.9645	0.0875	0.0875	0.0875	0.0425	0.0106	0.9519
(20,30)	30	(20,0$_{(29)}$)	0.0641	2.4755	0.9575	0.0829	0.0829	0.0829	0.0758	0.0062	0.9255
		(0$_{(19)},20,$0$_{(10)}$)	0.0712	2.1912	0.9603	0.0867	0.0867	0.0867	0.0767	0.0058	0.9338
		(0$_{(29)},20)$	0.0569	3.1415	0.9565	0.0572	0.0572	0.0572	0.0501	0.0079	0.9485
(30,20)	30	(20,0$_{(29)}$)	0.0762	3.0096	0.9440	0.0762	0.0762	0.0762	0.0652	0.008	0.9472
		(0$_{(19)},20,$0$_{(10)}$)	0.0695	2.7421	0.9405	0.0691	0.0691	0.0691	0.0541	0.0069	0.9668
		(0$_{(29)},20)$	0.0692	3.7951	0.9351	0.0693	0.0693	0.0693	0.0543	0.0093	0.9414
(30,20)	40	(10,0$_{(39)})$	0.0752	2.664	0.9564	0.0745	0.0745	0.0745	0.0655	0.0069	0.9743
		(0$_{(19)},10,0_{(20)}$)	0.0656	2.4538	0.9528	0.0658	0.0658	0.0658	0.0598	0.0063	0.9256
		(0$_{(39)},10$)	0.0786	3.1684	0.9659	0.0785	0.0785	0.0785	0.0695	0.0085	0.9423

**Table 9:** The comparison between A.E.B and A.E.A.

$\left( n,m\right)$	*r*	($R_{1,.....,}R_{r}$)	p = 0.39		p = 0.61	
			A.E.B	A.E.A	A.E.B	A.E.A
$\left( 10,10\right)$	5	(0, 0, 0, 0, 15)	2.85343	3.045	3.85343	2.012
(5,15)			3.99969	4.084	4.969	0.861
(15,5)			1.53201	1.615	2.3201	3.35
(10,10)	5	(15, 0, 0, 0, 0)	2.63847	2.681	4.63847	2.362
(5,15)			3.86393	3.843	5.86393	1.158
(15,5)			1.34412	1.385	2.16385	3.664
(10,10)	5	(6,0,4,0,5)	2.92725	2.915	2.9222	2.103
(5,15)			4.05637	4.022	5.5637	0.973
(15,5)			1.64331	1.575	2.4321	3.463
$\left( 10,10\right)$	10	(0$_{(9)},10$)	5.573	5.525	6.953	4.441
(5,15)			7.91699	7.908	8.699	2.095
(15,5)			2.94128	2.916	3.418	7.047
$\left( 10,10\right)$	10	(10,0$_{(9)}$)	5.11094	5.085	6.098	4.891
(5,15)			7.60506	7.522	8.506	2.405
(15,5)			2.56783	2.576	3.783	7.402
(20,20)	20	(0$_{(19)},20$)	11.1254	11.207	12.254	8.824
(15,25)			13.5408	13.583	15.131	10.1931
(25,15)			8.56862	8.548	9.2359	6.2359
(20,20)	20	(20,0$_{(19)})$	10.1024	10.088	10.241	9.916
(15,25)			12.6126	12.584	13.3126	7.336
(25,15)			7.58382	7.587	8.382	12.425
(30,30)	30	(0$_{(29)},30$)	16.678	16.814	18.068	13.363
(20,40)			21.465	21.579	20.445	18.516
(40,20)			11.5161	11.457	12.561	18.506
(30,30)	30	(30,0$_{(29)})$	15.0975	15.067	16.0215	14.975
(20,40)			20.1109	20.107	18.1109	10.927
(40,20)			10.0718	10.111	11.718	20.022
(40,40)	40	(0$_{(39)},40$)	22.2307	22.389	23.807	17.635
(30,50)			27.0638	27.117	28.538	12.913
(50,30)			17.117	17.155	19.017	22.843
(40,40)	40	(40,0$_{(39)})$	20.0939	20.169	21.239	19.899
(30,50)			25.1069	25.065	26.469	14.927
(50,30)			15.0757	15.015	16.7257	24.934

## Conclusion

In this study, a joint type-II progressively censoring method was used to investigate two samples with a power Rayleigh distribution. It was believed that the scale parameters and form parameters were different. The MLE estimates were obtained using the maximum likelihood method. Performance of MLEs and Bayesian estimation methods were compared for informative and non-informative priors. Importance sampling was used to create Bayesian estimates. It was also investigated how estimates under square and LINEX loss functions compared. The best method for point estimates found out to be Bayesian inference under informative priors, and a number of censoring scheme structures were found. On a real data set, we have used the developed techniques. A simulation study is used to compare the performance of the proposed methods for different sample sizes (*m*, *n*). From the results, we observe the following:

1. . From Table 2, it can be seen that when $c=2$, the Bayes estimates under the SE loss function are similar to those under the LINEX loss functions.
2. . It is observed that, from Table 3 the MCMC is better than the MLE in the sense of having the smallest lengths.
3. . It is clear from Table 4 the values of A.E.B. are smaller than the values of M.E.A. in all schemes.
4. . It can be seen from Table 9 the values of A.E.B. are relatively close to the values of M.E.A. in all schemes.
5. . It is evident that from Tables 5, 6, 7, and 8 the MSEs and CP of MLE are smaller than the MSEs of MCMC. Then, the performance of the Bayes estimates for the parameters $\alpha _{1},\ \alpha _{2}~, \beta _{1}$ and $\ \beta _{2}$ are better than the MLEs.
6. . It is observed that from Tables 5, 6, 7, and 8 the Bayes estimates under LINEX with $c=2$ are provides better estimates in the sense of having smaller MSEs
7. . It is clear that from Tables 5, 6, 7 , and 8 when *m*, *n* and *r* increase the MSEs and the lengths decrease
8. . It is evident from Tables 5, 6, 7, and 8 that the MCMC CRIs give more accurate results than the ACIs since the lengths of the MCMC CRIs are less than the lengths of the ACIs, for various sample sizes.

## Data Availability

Data are available in paper.
